# Drug-Eluting Bead Bronchial Arterial Chemoembolization With and Without Microwave Ablation for the Treatment of Advanced and Standard Treatment-Refractory/Ineligible Non-Small Cell Lung Cancer: A Comparative Study

**DOI:** 10.3389/fonc.2022.851830

**Published:** 2022-03-15

**Authors:** Sheng Xu, Zhi-Xin Bie, Yuan-Ming Li, Bin Li, Fan-Lei Kong, Jin-Zhao Peng, Xiao-Guang Li

**Affiliations:** ^1^Department of Minimally Invasive Tumor Therapies Center, Beijing Hospital, National Center of Gerontology, Institute of Geriatric Medicine, Chinese Academy of Medical Sciences, Beijing, China; ^2^Graduate School of Peking Union Medical College, Chinese Academy of Medical Sciences, Beijing, China

**Keywords:** microwave ablation, non-small cell lung cancer, drug-eluting beads, chemoembolization, complications, survival

## Abstract

**Purpose:**

To compare the outcomes of drug-eluting bead bronchial arterial chemoembolization (DEB-BACE) with and without microwave ablation (MWA) for the treatment of advanced and standard treatment-refractory/ineligible non-small cell lung cancer (ASTRI-NSCLC).

**Materials and Methods:**

A total of 77 ASTRI-NSCLC patients who received DEB-BACE combined with MWA (group A; n = 28) or DEB-BACE alone (group B; n = 49) were included. Clinical outcomes were compared between groups A and B. Kaplan–Meier methods were used to compare the median progression-free survival (PFS) or overall survival (OS) between the two groups. Univariate and multivariate Cox proportional hazards analyses were used to investigate the predictors of OS for ASTRI-NSCLC treated with DEB-BACE.

**Results:**

No severe adverse event was found in both groups. Pneumothorax was the predominant MWA-related complication in group A, with an incidence rate of 32.1% (9/28). Meanwhile, no significant difference was found in DEB-BACE-related complications between groups A and B. The overall disease control rate (DCR) was 61.0% (47/77), with a significantly higher DCR in group A (85.7% vs. 46.9%, *P* = 0.002). The median PFS in groups A and B was 7.0 and 4.0 months, respectively, with a significant difference (*P* = 0.037). The median OS in groups A and B was both 8.0 months, with no significant difference (*P* = 0.318). The 6-month PFS and OS rates in groups A and B were 75.0% and 78.6%, 22.4% and 59.2%, respectively, while the 12-month PFS and OS rates in groups A and B were 17.9% and 28.6%, 14.3% and 22.4%, respectively. Of these, a significantly higher 6-month PFS rate was found in group A (75.0% vs. 22.4%; *P <* 0.001). The cycles of DEB-BACE/bronchial artery infusion chemotherapy [hazard ratio (HR): 0.363; 95% confidence interval (CI): 0.202–0.655; *P* = 0.001] and postoperative immunotherapy (HR: 0.219; 95% CI: 0.085–0.561; *P* = 0.002) were identified as the predictors of OS in ASTRI-NSCLC treated with DEB-BACE.

**Conclusion:**

MWA sequentially combined with DEB-BACE was superior to DEB-BACE alone in the local control of ASTRI-NSCLC. Although the combination therapy reveals a trend of prolonging the OS, long-term prognosis warrants an investigation with a longer follow-up.

## Introduction

Primary lung cancer has an incidence and mortality of over 2.2 million and 1.79 million, respectively, estimated globally in 2020 (1). In China, non-small cell lung cancer (NSCLC) has a percentage over 85% in the diagnoses of lung cancer, and most of the patients are advanced when diagnosed (2, 3). The standard treatments for unresectable advanced NSCLC include chemoradiotherapy, molecular targeted therapy [such as tyrosine kinase inhibitors (TKIs)], and immunotherapy. However, it often develops refractoriness and almost 59% of unresectable stage III NSCLC patients are ineligible for chemoradiotherapy owing to the severe adverse events (4). In addition, TKIs are not available for a large proportion of patients without gene mutations. The prognosis of advanced NSCLC remains dismal, with a 5-year survival rate of 15% for stage III and that of less than 10% for stage IV (5). Furthermore, 30%–50% of NSCLC patients are diagnosed with poor performance status owing to cardiovascular and pulmonary diseases and are ineligible for systemic therapy (6). The therapeutic strategy for advanced and standard treatment-refractory/ineligible NSCLC (ASTRI-NSCLC) patients remains limited.

In recent decades, computed tomography (CT)-guided microwave ablation (MWA) has been recommended as a primary treatment option by several guidelines (7, 8), especially for early-stage NSCLC that is contraindicated to undergo surgery or radiotherapy, and has been presented as a part of combination therapy for advanced-stage NSCLC (9). Moreover, bronchial arterial chemoembolization (BACE) has been increasingly applied in NSCLC, which aims to embolize the tumor-feeding arteries, extend the action time of chemotherapeutic drugs, and increase drug concentration at tumor tissues (10). As a novel drug delivery and embolization system in transarterial chemoembolization (TACE), drug-eluting bead (DEB) microsphere can release the loaded chemotherapeutic drugs slowly, improve local drug concentration, and permanently embolize the tumor-feeding arteries (11). DEB-BACE was identified as an effective and safe approach for advanced standard treatment-ineligible/rejected NSCLC (12–16).

The optimal cutoff value of tumor diameter in predicting local recurrence after MWA was 3 cm, with the potential mechanisms for incomplete ablation in large tumors including the limited size and homogeneity of tumor necrosis achieved by MWA and the heat-sink effect caused by the blood vessels in ablated tumor (17). In theory, BACE could block the arterial blood flow and increase drug concentration at tumor sites, which may improve the prognosis of MWA. Similar effectiveness has already been shown and recommended in liver cancers treated with TACE and thermal ablation (18, 19). Liu et al. (20) indicate that combined treatment with DEB-TACE has advantages in improving the prognosis of early-stage liver cancer compared to MWA alone. However, few studies have focused on the outcomes of MWA combined with DEB-BACE for ASTRI-NSCLC. Therefore, a retrospective study was conducted to compare the outcomes of DEB-BACE with and without MWA in the treatment of ASTRI-NSCLC.

## Materials and Methods

### Patient Criteria

This single-center case-control study included all ASTRI-NSCLC patients who underwent DEB-BACE at this institution. The institutional ethics review board approved this study. The study protocol was conducted per the Declaration of Helsinki. Informed consent was waived due to the retrospective nature of this study. ASTRI-NSCLC patients who received DEB-BACE between May 2017 and April 2021 were allocated to the combination therapy group (MWA+DEB-BACE, group A) and DEB-BACE alone group (group B). Informed consent of MWA or/and DEB-BACE was obtained before these procedures. Patient inclusion criteria were as follows: (a) age of 18–85 years; (b) advanced NSCLC that is ineligible for or is refractory to standard treatments; and (c) Eastern Cooperation Oncology Group score of 0–2. Patient exclusion criteria were as follows: (a) concomitant radioactive seed implantation was performed during MWA procedure; (b) incomplete data; (c) lost to follow-up; (d) MWA combined with bronchial artery infusion chemotherapy (BAI); and (e) period between MWA and DEB-BACE longer than 1.5 months.

The histopathological subtypes were confirmed *via* previous percutaneous needle aspiration biopsy or surgery or fiberoptic bronchoscopy. Positron emission tomography or CT was performed to evaluate the tumor location, quantity, size, and local and distant metastases. The clinical TNM staging system of the Union for International Cancer Control (8th edition) was applied in identifying the tumor stage (5).

### Microwave Ablation Procedure

The MWA indications and procedures followed the Society of Interventional Radiology (SIR) guidelines (21). As described previously (22), an MTC-3C MWA system (Vison Medical Inc., China) or an ECO-100A1 MWA system (ECO, China) was used, with a microwave emission frequency of 2,450 ± 50 MHz and an adjustable continuous wave output power of 20–80 W. The MWA antenna (Vison or ECO) was 15–18 cm in effective length and 15–17 G in outside diameter according to the tumor location and distance to the pleura, with a 15-mm active tip. Preprocedural CT was performed to inform the treatment plan and to clarify the suitable position, puncture site location, optimal puncture trajectory, and the number of MWA antennas. Local anesthesia was used for most patients, while intravenous anesthesia was used for selected patients requiring more pain control. Antennas were introduced into the planned site, and MWA was performed at the planned power and duration, with adjustments of suitable power and duration being carried out according to the intraprocedural location of MWA antennas as needed. The procedure was terminated when the tumor tissues were ablated and inactivated as much as possible. Finally, chest CT was repeated to evaluate the distribution of ablation zone and detect the potential complications.

### DEB-BACE Procedure

As described previously (16), DEB-BACE procedures were performed under local anesthesia *via* a femoral artery approach. A 5-French pigtail catheter (PIG Impress; Merit Medical Systems, Inc., USA) was initially used for aortic arch angiography to detect the origins of tumor-feeding arteries. Then, a 5-French cobra (CB 1 Impress; Merit Medical Systems, Inc., USA) or left gastric catheter (Radifocus; Terumo Corporation, Japan) was used to select the bronchial arteries or non-bronchial systemic arteries. Super-selective catheterization with a 1.98-French microcatheter (Masters PARKWAY SOFT; Asahi Intec Co., Japan) was inserted coaxially. The chemotherapeutic regimen of paclitaxel (100–200 mg; Keaili, CSPC Pharmaceutical Group Co., China) was administered during BAI for patients who had already received systemic platinum-based chemotherapy, while nedaplatin (80–100 mg; Lubei, Qilu Pharmaceutical Co., China) was administered for patients who had not received systemic platinum-based chemotherapy. Gemcitabine (200–1,000 mg; Zefei, Hansoh Pharmaceutical Group Co., China) or endostatin (60-120 mg; Endu, Simcere Pharmaceutical Group, China) was infused on demand. BAI was followed by DEB-BACE, which was performed using 100–300 μm or 300–500 μm or 500–700 μm CalliSpheres microspheres (Jiangsu Hengrui Medical Co., China) loaded with gemcitabine (800 mg; Hansoh, China) or pirarubicin (30 mg; Adriamycin, Shenzhen Main Luck Pharmaceutical Inc., China). The CalliSpheres microspheres were first mixed with gemcitabine or pirarubicin at a temperature of 23°C–28°C for 30 min and vibrated every 5 min. Then, iodixanol (100 ml:65.2 g/32 g iodine; Hengrui, China) was added at a 1:1 ratio. The DEB-BACE was performed slowly and carefully in tumor-feeding arteries under fluoroscopic monitoring to avoid reflux into nontarget vessels. The technical endpoint of embolization was the absence of additional tumor staining or stasis/near stasis of the tumor-feeding arteries. For patients who received combination therapy, DEB-BACE was performed 2–45 days after MWA, followed by repeated DEB-BACE/BAI on demand. For the patients who received a repeated DEB-BACE/BAI, DEB-BACE was performed if abundant tumor staining was revealed in angiography, while BAI alone was performed for those patients without.

### Follow-Up and Assessments

Short-term follow-up with CT was conducted 1–5 days after MWA during hospitalization and 3–4 weeks after MWA at an outpatient visit to detect postprocedural complications, including pneumothorax and pleural effusion. Chest tube placement was performed for patients with moderate and severe pneumothorax or pleural effusion (17). Immunotherapy was performed for patients who have not received and can tolerate programmed cell death ligand 1 (PD-L1) blockade and programmed cell death 1 (PD-1) blockade. PD-L1 blockade was administered for patients with high PD-L1 expression according to the previous testing.

MWA- or DEB-BACE-related complications were evaluated according to criteria from the SIR (21, 23). Enhanced CT was routinely performed every 3 months. Based on the Response Evaluation Criteria in Solid Tumors version 1.1 (24), treatment response was evaluated and classified as a complete response (CR), partial response (PR), stable disease (SD), or progressive disease (PD). The disease control rate (DCR) was defined as CR or PR or SD. Overall survival (OS) was defined as the interval from the start of MWA in group A or the first DEB-BACE in group B to death or the last follow-up (October 31, 2021). For patients who died during the follow-up period, OS was calculated as the interval from the MWA or DEB-BACE procedures to death. For patients who survived but were lost to follow-up, OS was calculated as the interval from the MWA or DEB-BACE procedures to the last follow-up. Progression-free survival (PFS) was defined as the interval from the MWA or DEB-BACE procedures to the time of objective progression, including local progression and/or distant metastases. For patients who did not die or progress, the censoring date was defined as the last clinical assessment date.

### Statistical Analysis

Categorical variables are described as frequencies and percentages, and continuous variables are described as the mean ± SD. Statistical analyses were performed using SPSS 25.0 for Windows (IBM, Somers, New York). The clinical characteristics, complications, and prognostic data were compared by Student’s t-test or Mann–Whitney U test for continuous variables and by chi-square test for categorical variables. Of these, Kaplan–Meier methods were used to compare the median PFS or OS between the two groups. The OS for ASTRI-NSCLC treated with DEB-BACE was estimated using the Kaplan–Meier method. The possible predictors of OS included 21 parameters on demographics, treatment history, MWA or/and DEB-BACE factors, and radiological features. Variables with a *P*-value <0.05 in the univariate analyses were entered as candidate variables into a stepwise Cox proportional hazards analyses. The results of the multivariate analyses indicated the predictors for the OS. A *P*-value <0.05 was considered to indicate statistical significance in the univariate and multivariate Cox proportional hazards analyses.

## Results

### Patient Characteristics

**Figure 1:**
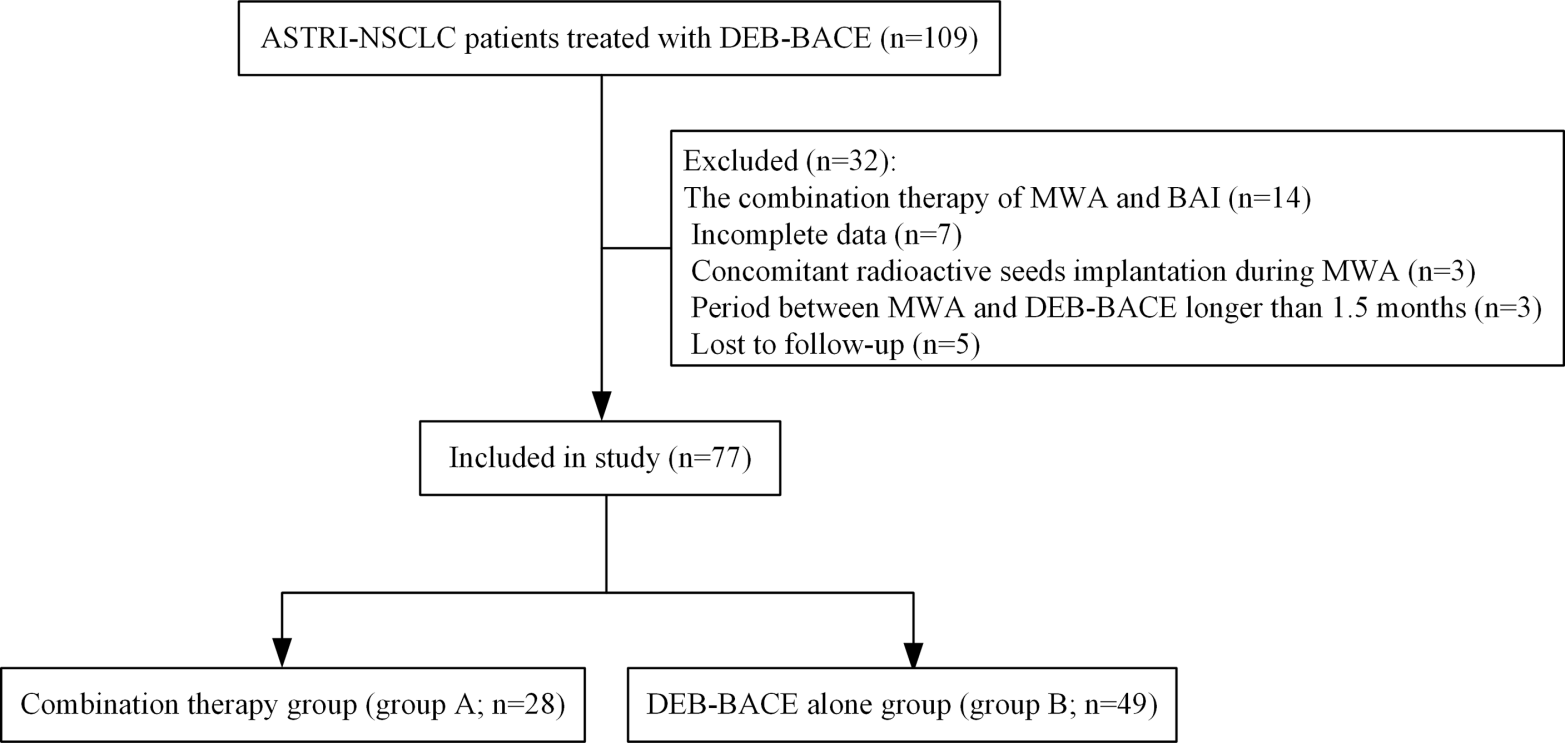
Patient selection flowchart. ASTRI-NSCLC, advanced standard treatment-refractory/ineligible non-small cell lung cancer; DEB-BACE, drug-eluting bead bronchial artery chemoembolization; MWA, microwave ablation; BAI, bronchial artery infusion chemotherapy.

A total of 77 NSCLC patients (28 in group A and 49 in group B; **Figure 1**) were included. Of these, 41 patients (53.2%) were squamous cell carcinoma and 45 patients (58.4%) were stage III NSCLC. Detailed demographic characteristics are presented in **Table 1**; a significant difference was found in the incidence of hypertension (*P* = 0.021), whereas other variables revealed no differences. There were 21 patients (27.3%) who received immunotherapy after DEB-BACE, with PD-L1 blockade administered in six patients (7.8%) and PD-1 blockade administered in 15 patients (19.5%).

**Table 1:** Clinical characteristics between ASTRI-NSCLC patients in groups A and B.

Variables	Overall (n = 77)	Group A (n = 28)	Group B (n = 49)	*P*-value
Age (years)	67.6 ± 9.8	66.9 ± 8.9	68.0 ± 10.4	0.637
Gender				0.219
Men	57 (74.0%)	23 (82.1%)	34 (69.4%)	
Women	20 (26.0%)	5 (17.9%)	15 (30.6%)	
Comorbidity				
Hypertension	26 (33.8%)	5 (17.9%)	21 (42.9%)	0.026
Diabetes	11 (14.3%)	3 (10.7%)	8 (16.3%)	0.735
Cardiocerebrovascular diseases	17 (22.1%)	6 (21.4%)	11 (22.4%)	0.917
Pulmonary diseases	6 (7.8%)	2 (7.1%)	4 (8.2%)	>0.999
Tumor subtypes				0.995
Adenocarcinoma	28 (36.4%)	10 (35.7%)	18 (36.7%)	
Squamous cell carcinoma	41 (53.2%)	15 (53.6%)	26 (53.1%)	
Others	8 (10.4%)	3 (10.7%)	5 (10.2%)	
Tumor stage				0.760
III	45 (58.4%)	17 (60.7%)	28 (57.1%)	
IV	32 (41.6%)	11 (39.3%)	21 (42.9%)	
Treatment history				
Previous surgery	5 (6.5%)	2 (7.1%)	3 (6.1%)	>0.999
Previous chemotherapy	24 (31.2%)	5 (17.9%)	19 (38.8%)	0.057
Previous radiotherapy	10 (13.0%)	3 (10.7%)	7 (14.3%)	0.654
Previous TKIs	15 (19.5%)	3 (10.7%)	12 (24.5%)	0.242
Radiological features				
Tumor diameter (cm)	6.3 ± 2.6	6.2 ± 2.4	6.4 ± 2.8	0.737
Location				0.208
Lower or middle lobe	34 (44.2%)	15 (53.6%)	19 (38.8%)	
Upper lobe	43 (55.8%)	13 (46.4%)	30 (61.2%)	
Emphysema	20 (26.0%)	10 (35.7%)	10 (20.4%)	0.141
Extrapulmonary metastases	20 (26.0%)	6 (21.4%)	14 (28.6%)	0.492
Tumor number				0.285
1	65 (84.4%)	22 (78.6%)	43 (87.8%)	
≥2	12 (15.6%)	6 (21.4%)	6 (12.2%)	
Laboratory examinations				
WBC (*10^9^/L)	7.8 ± 2.6	8.2 ± 2.8	7.6 ± 2.5	0.294
Hb (g/L)	121.4 ± 18.4	125.9 ± 17.5	118.8 ± 18.5	0.107
PLT (*10^9^/L)	265.2 ± 89.8	279.0 ± 89.1	257.4 ± 90.1	0.311
PT (s)	11.7 ± 1.6	11.5 ± 2.2	11.8 ± 1.1	0.377
Postoperative treatments				
TKIs	21 (27.3%)	5 (17.9%)	16 (32.7%)	0.161
Immunotherapy	21 (27.3%)	10 (35.7%)	11 (22.4%)	0.209
MWA-related factors				
Number of MWA antennas	/	1.4 ± 0.6	/	/
Maximum power (W)	/	51.8 ± 13.3	/	/
Ablation time (min)	/	12.6 ± 7.0	/	/
Number of pleural punctures	/	1.8 ± 0.8	/	/
Diameter of instruments				
15G	/	15 (53.6%)	/	/
17G	/	13 (46.4%)	/	/
DEB-BACE-related factors				
Diameter of microsphere (μm)				>0.999
100–300	13 (16.9%)	5 (17.9%)	8 (16.3%)	
300–500	62 (80.5%)	22 (78.6%)	40 (81.6%)	
500–700	2 (2.6%)	1 (3.6%)	1 (2.0%)	
Loaded drug				>0.999
Gemcitabine	69 (89.6%)	25 (89.3%)	44 (89.8%)	
Pirarubicin	8 (10.4%)	3 (10.7%)	5 (10.2%)	
BAI drugs				
Gemcitabine	34 (44.2%)	13 (46.4%)	21 (42.9%)	0.761
Platinum	52 (67.5%)	19 (67.9%)	33 (67.3%)	0.963
Endostatin	44 (57.1%)	20 (71.4%)	24 (49.0%)	0.056
Paclitaxel	21 (27.3%)	11 (39.3%)	10 (20.4%)	0.074
Embolized arteries				0.055
Bronchial artery	67 (87.0%)	27 (96.4%)	40 (81.6%)	
NBSA	6 (7.8%)	1 (3.6%)	5 (10.2%)	
Bronchial artery+NBSA	4 (5.2%)	0	4 (8.2%)	
Number	1.1 ± 0.4	1.1 ± 0.3	1.2 ± 0.4	0.137
DEB-BACE/BAI cycles	2.0 ± 1.3	1.9 ± 1.1	2.0 ± 1.4	0.722
Cycles of combination therapy	/	1.1 ± 0.4	/	/
Period between MWA and DEB-BACE (days)	/	15.5 ± 14.7	/	/

Frequencies and percentages are reported for categorical variables, and mean ± SD are reported for continuous variables.

ASTRI-NSCLC, advanced standard treatment-refractory/ineligible non-small cell lung cancer; MWA, microwave ablation; DEB-BACE, drug-eluting bead bronchial artery chemoembolization; WBC, white blood cell; Hb, hemoglobin; PLT, platelet; PT, prothrombin time; TKIs, tyrosine kinase inhibitors; MWA, microwave ablation; BAI, bronchial artery infusion chemotherapy; NBSA, non-bronchial systemic artery.

### Complications

No severe adverse event was found in both groups. Detailed MWA-related complications in group A are presented in **Table 2**; pneumothorax was the predominant MWA-related complication, with an incidence rate of 32.1% (9/28). Detailed DEB-BACE-related complications between groups A and B are presented in **Table 3**, with no significant difference.

**Table 2:** Details of MWA-related complications in group A.

Variables	Number	Percentage (%)
Major complications		
Pneumothorax	5	17.9
Pleural effusion	1	3.6
Bronchopleural fistula	0	0.0
Pneumonia	0	0.0
Minor complications		
Pneumothorax	4	14.3
Pneumonia	1	3.6
Side effects		
Chest pain	5	17.9
Pleural effusion	1	3.6
Post-ablation syndrome	6	21.4

MWA, microwave ablation.

**Table 3:** Details of DEB-BACE-related complications between ASTRI-NSCLC patients in groups A and B.

Variables	Overall (n = 77)	Group A (n = 28)	Group B (n = 49)	*P*-value
Mild adverse event				
Chest congestion or pain	8 (10.4%)	3 (10.7%)	5 (10.2%)	>0.999
Fever	5 (6.5%)	2 (7.1%)	3 (6.1%)	>0.999
Vomit	1 (1.3%)	1 (3.6%)	0	0.364
Moderate adverse event				
Chest congestion or pain	4 (5.2%)	1 (3.6%)	3 (6.1%)	>0.999
Fever	5 (6.5%)	1 (3.6%)	4 (8.2%)	0.760
Myelosuppression	3 (3.9%)	1 (3.6%)	2 (4.1%)	>0.999
Severe adverse event	/	/	/	/
Life-threatening or disabling event	/	/	/	/
Patient death or unexpected pregnancy abortion	/	/	/	/

DEB-BACE, drug-eluting bead bronchial artery chemoembolization.

### Clinical Outcomes

**Figure 2:**
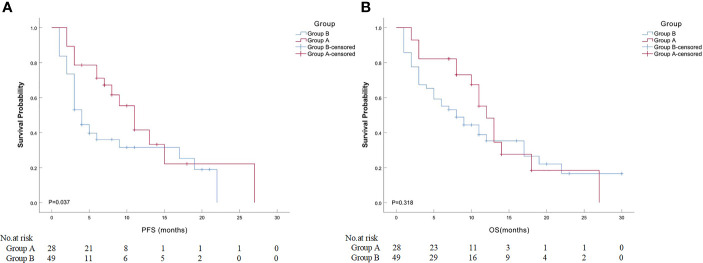
Comparison of median PFS or OS between ASTRI-NSCLC patients in groups A and B. **(A)** The estimated median PFS was 11.0 months for patients in group A, while that was 4.0 months for patients in group B. **(B)** The estimated median OS was 12.0 months for patients in group A, while that was 8.0 months for patients in group **(B)** PFS, progression-free survival; OS, overall survival; ASTRI-NSCLC, advanced standard treatment-refractory/ineligible non-small cell lung cancer.

**Figure 3:**
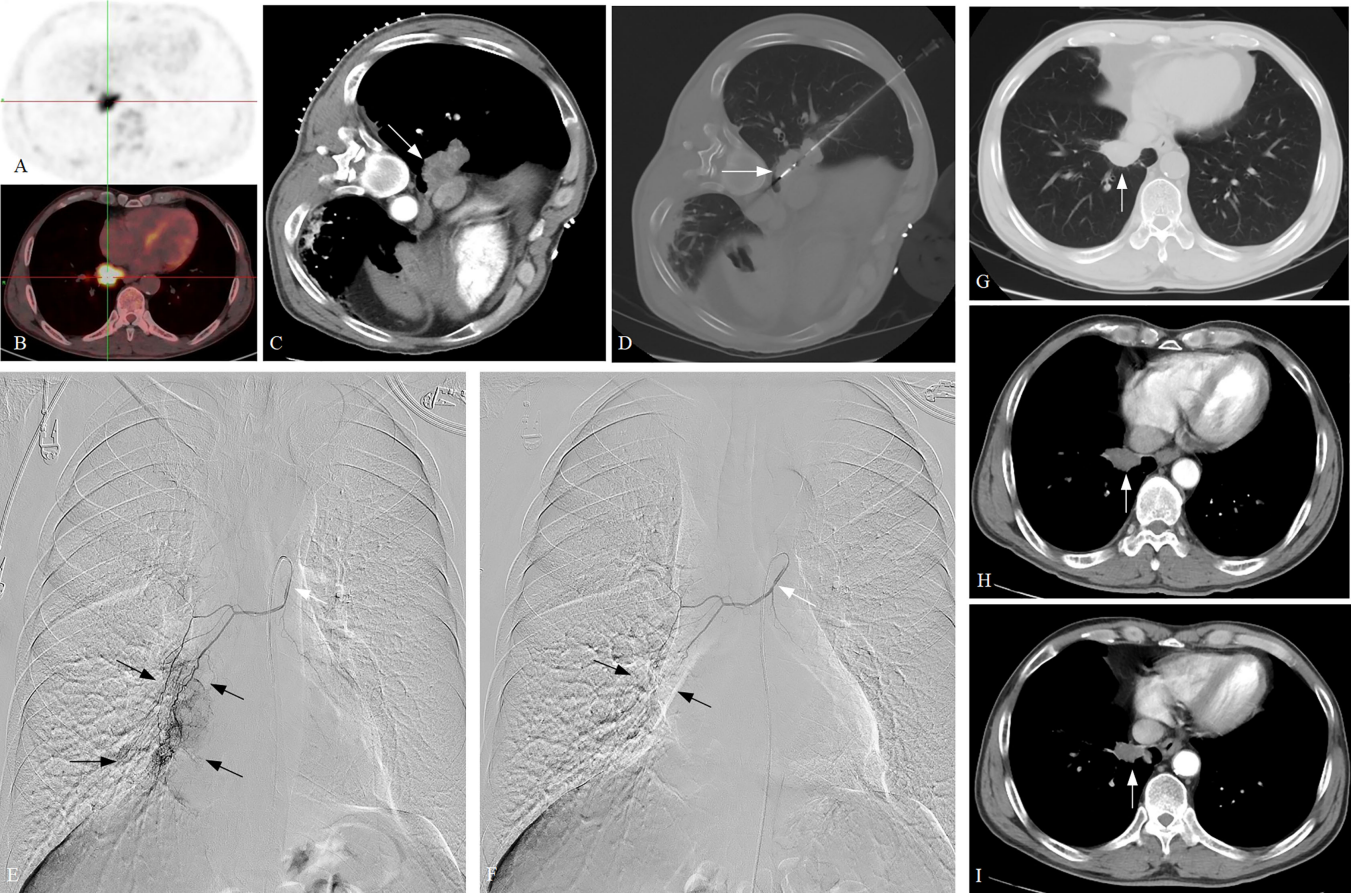
A typical case of ASTRI-NSCLC treated with combination therapy. **(A–C)** A confirmed NSCLC patient with poor pulmonary function was admitted, with the tumor subtype of squamous cell carcinoma. Positron emission tomography revealed the presence of abnormal accumulation of the tracer in lung mass and enlarged hilar lymph nodes, and the maximum tumor diameter was 5.1 cm (white arrow), which showed T3N1M0 and stage III A for the patient. **(D)**. CT-guided MWA was performed (white arrow), with 40W of energy released and 10 minutes of ablation time. **(E)** DEB-BACE was performed 3 weeks after MWA, with the microcatheter being used for super-selective catheterization initially (white arrow). Sequential angiography revealed that the tumor was fed by the right bronchial artery, with the presence of abundant tumor staining (black arrow). **(F)** The 300–500-μm CalliSpheres microspheres loaded with gemcitabine (800 mg) were used for chemoembolization *via* the microcatheter (white arrow). The post-embolization angiography revealed the disappearance of tumor staining (black arrow). A total of four cycles of DEB-BACE/BAI were performed. **(G)** The 3-month CT scan after combination therapy revealed the tumor size decreases to 3.5 cm and showed a PR in response. **(H, I)** The 6-month and 9-month CT scans after combination therapy revealed a continued decrease in the tumor size. ASTRI-NSCLC, advanced standard treatment-refractory/ineligible non-small cell lung cancer; CT, computed tomography; MWA, microwave ablation; DEB-BACE, drug-eluting bead bronchial artery chemoembolization; BAI, bronchial artery infusion chemotherapy; PR, partial response.

Detailed clinical outcomes between the two groups are presented in **Table 4**. In a mean follow-up of 21.7 ± 14.1 months, the median PFS in groups A and B was 7.0 and 4.0 months, respectively, with a significant difference (*P* = 0.037; **Figure 2A**). The median OS in groups A and B was both 8.0 months, with no significant difference (*P* = 0.318; **Figure 2B**). The 6-month PFS and OS rates in groups A and B were 75.0% and 78.6%, 22.4% and 59.2%, respectively, while the 12-month PFS and OS rates in groups A and B were 17.9% and 28.6%, 14.3% and 22.4%, respectively. Of these, a significantly higher 6-month PFS rate was found in group A (75.0% vs. 22.4%; *P* < 0.001). There were nine patients (11.7%) who achieved PR at 3 months after MWA or/and DEB-BACE (**Figure 3**). The overall DCR was 61.0% (47/77) at 3 months after MWA or DEB-BACE; of these, a significantly higher DCR was found in group A (85.7% vs. 46.9%, *P* = 0.002).

**Table 4:** Clinical outcomes between ASTRI-NSCLC patients in groups A and B.

Variables	Overall (n = 77)	Group A (n = 28)	Group B (n = 49)	*P*-value
Response				0.002
CR	/	/	/	
PR	9 (11.7%)	5 (17.9%)	4 (8.2%)	
SD	38 (49.4%)	19 (67.9%)	19 (38.8%)	
PD	30 (39.0%)	4 (14.3%)	26 (53.1%)	
DCR (%)	61.0 (47/77)	85.7 (24/28)	46.9 (23/49)	0.002
Status				0.279
Survival	27 (35.1%)	12 (42.9%)	15 (30.6%)	
Death	50 (64.9%)	16 (57.1%)	34 (69.4%)	
6-month PFS rate (%)	41.6 (32/77)	75.0 (21/28)	22.4 (11/49)	<0.001
12-month PFS rate (%)	15.6 (12/77)	17.9 (5/28)	14.3 (7/49)	0.678
6-month OS rate (%)	66.2 (51/77)	78.6 (22/28)	59.2 (29/49)	0.084
12-month OS rate (%)	24.7 (19/77)	28.6 (8/28)	22.4 (11/49)	0.549

ASTRI-NSCLC, advanced standard treatment-refractory/ineligible non-small cell lung cancer; MWA, microwave ablation; DEB-BACE, drug-eluting bead bronchial artery chemoembolization; CR, complete response; PR, partial response; SD, stable disease; PD, progression disease; DCR, disease control rate; PFS, progression-free survival; OS, overall survival.

### Predictors of Overall Survival for ASTRI-NSCLC Treated With DEB-BACE

**Figure 4:**
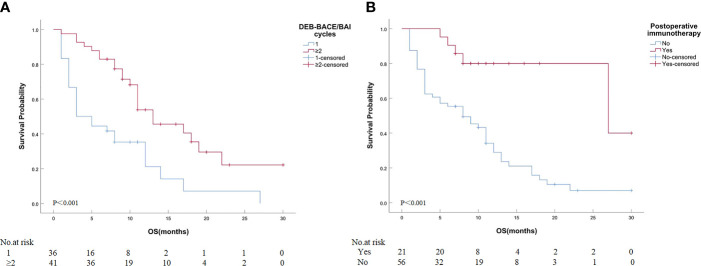
Kaplan–Meier analyses of OS in ASTRI-NSCLC treated with DEB-BACE. **(A)** The estimated median OS was 3.0 months for patients treated with one cycle of DEB-BACE compared with 13.0 months for those patients with no less than two cycles of DEB-BACE/BAI. **(B)** The estimated median OS was 27.0 months for patients with postoperative immunotherapy compared with 8.0 months for those patients without. OS, overall survival; ASTRI-NSCLC, advanced standard treatment-refractory/ineligible non-small cell lung cancer; DEB-BACE, drug-eluting bead bronchial artery chemoembolization; BAI, bronchial artery infusion chemotherapy.

Detailed results of univariate and multivariate analyses are presented in **Table 5**. The cycles of DEB-BACE/BAI [hazard ratio (HR): 0.363, 95% CI: 0.202–0.655, *P* = 0.001; **Figure 4A**] and postoperative immunotherapy (HR: 0.219, 95% CI: 0.085–0.561, *P* = 0.002; **Figure 4B**) were identified as the predictors of OS for ASTRI-NSCLC treated with DEB-BACE.

**Table 5:** Univariate and multivariate Cox proportional hazards analyses for OS in ASTRI-NSCLC treated with DEB-BACE.

Variables	Univariate analysis		Multivariate analysis	
	Median OS (95% CI)	*P*-value*	HR (95% CI)	*P*-value**
Tumor stage		0.030		
III	12.0 (8.863–15.137)			
IV	6.0 (1.067–10.933)			
Previous radiotherapy		0.048		
Yes	3.0 (0.000–7.132)			
No	11.0 (9.189–12.811)			
Tumor number		0.026		
1	11.0 (7.273–14.727)			
≥2	3.0 (0.000–9.365)			
DEB-BACE/BAI cycles		<0.001		0.001
1	3.0 (0.795–5.205)		1	
≥2	13.0 (8.443–17.557)		0.363(0.202–0.655)	
Postoperative immunotherapy		<0.001		0.002
Yes	27.0 (0.335–53.665)		0.219 (0.085–0.561)	
No	8.0 (3.626–12.374)		1	

*Log-rank test was used.

**Cox proportional hazards regression analysis was used.

OS, overall survival; ASTRI-NSCLC, advanced standard treatment-refractory/ineligible non-small cell lung cancer; DEB-BACE, drug-eluting bead bronchial artery chemoembolization; CI, confidence interval; HR, hazard ratio; BAI, bronchial artery infusion chemotherapy.

## Discussion

The first-line therapeutic strategy for unresectable advanced NSCLC is chemoradiotherapy, which can provide an objective response rate (ORR) of 23%–34% and a median OS of 11.6 months after systemic platinum-based chemotherapy, or an ORR of 51.5%, a median OS of 22.0 months, and a median PFS of 17.0 months after chemoradiotherapy (25–27). The incidence rate of adverse events was 62% as reported (26). Second-line systemic chemotherapy or TKIs were considered when the patients fail to respond to chemoradiotherapy and can provide a median OS of 12.2 months and a median PFS of 3.1 months for stage III NSCLC patients (28).

In recent decades, BACE/BAI has been reported as an effective and safe approach for advanced NSCLC, especially for chemoradiotherapy-ineligible/rejected patients, with a median PFS of 6.5–14.0 months and a median OS of 13.1–25.0 months (29–31). NSCLC is mainly supplied by the bronchial artery (32), and the chemotherapeutic drug could be infused into the tumor-feeding arteries directly during BAI, which can improve the bioavailability of the drug without leading to severe adverse events (33). BAI has a local drug concentration 2–6 times higher than that of systemic chemotherapy and also has effects on lymph node metastases (34). Both local and systemic chemotherapy could be achieved in BACE/BAI, which attributes to the drugs entering the tumor again through the blood circulation, and tumor ischemia, necrosis, or shrinking caused by the embolization (35). In 2012, Nakanishi et al. (29) attempted BAI in 25 advanced chemotherapy-refractory/ineligible NSCLC patients and presented an ORR of 52% and a median PFS and OS of 6.5 months and 17.4 months, respectively. Similarly, another study performed 142 cycles of BAI in 40 advanced chemoradiotherapy-ineligible/rejected NSCLC patients and found an ORR of 32.5%, a DCR of 92.5%, and a median OS of 13.1 months (30). In 2017, Zhu et al. (31) analyzed 36 stage III squamous cell lung cancer patients treated with BAI and revealed a prolonged PFS or OS compared with systemic chemotherapy, with an effective rate, 1-year survival rate, and 2-year survival rate of 72.2%, 75.4%, and 52.1%, respectively.

DEB microspheres can provide a higher local drug concentration while reducing the systemic drug concentration and toxicity (11). Several studies have identified the efficacy and safety of DEB-BACE in advanced lung cancer and found a median PFS of 6.3–11.0 months and a median OS of 8.0–16.5 months (12–16). Gemcitabine-loaded DEB-BACE was first to be applied to NSCLC in 2019 (13). Although this study included only six patients, promising results with the median PFS of 8.0 months and median OS of 16.5 months were provided (13). Then, Shang et al. (12) compared the efficacy of pirarubicin-loaded DEB-BACE and BAI alone in 60 standard treatment-ineligible/rejected NSCLC patients; among them, over 85% of NSCLC patients were in advanced stage. Higher ORR, DCR, and 6-month PFS and OS rates were found in the DEB-BACE group, which indicated a superior efficacy than BAI alone (12). Sequentially, Zeng et al. (14) analyzed 23 advanced lung cancer patients treated with DEB-BACE and revealed an ORR of 78.3% and median OS of 15.6 months despite including four patients with small cell lung cancer or lung metastases. Another study from this institution showed median PFS and OS of 6.3 months and 10.2 months, respectively, for 29 advanced NSCLC patients treated with pirarubicin-loaded DEB-BACE (15). Nevertheless, there was no consensus on the optimal chemotherapeutic drug loaded in the DEB microsphere. The predominant chemotherapeutic drug loaded in the DEB microsphere was gemcitabine in this study. Although pirarubicin showed moderate anticancer activity, mild toxicity, and a high response rate for advanced NSCLC as reported (34), no superior efficacy of pirarubicin over gemcitabine was found in this study. The median PFS and OS of advanced NSCLC treated with DEB-BACE in this study were 5.0 and 8.0 months, which seem to be less than the reported results. Potential mechanisms were (a) over 40% of the patients were stage IV and over 50% of the patients were standard treatment-refractory, and (b) 35.1% of the patients survived until the last follow-up. In addition, the immune checkpoint inhibitors were identified as the second-line treatment for advanced NSCLC, and PD-1 blockade combined with systemic chemotherapy has been revealed as a promising approach (36). Identically, this study revealed that postoperative immunotherapy was one of the predictors to prolong the OS of ASTRI-NSCLC treated with DEB-BACE, which is per the additional immunotherapy that may improve the survival of advanced NSCLC after BAI/DEB-BACE, as reported (16).

The mechanisms of MWA are that microwaves can induce the oscillation of the water molecules that flip back and forth at a speed of 2–5 billion times a second depending on the frequency of the wave itself, allowing for a superior convection profile and causing coagulative necrosis conforming to the target sites (37). The optimal indication for thermal ablation is NSCLC with tumor <3 cm in diameter (8). Tumor diameter is a critical risk factor of local recurrence and OS after MWA in NSCLC as reported, especially for the tumor ≥3 cm in diameter (17, 38). Zhong et al. (39) indicated a local recurrence rate of 20.5% in advanced NSCLC treated with MWA; of these, 81.3% of relapse occurred in tumor size ≥3 cm, while Nelson et al. (40) summarized a local recurrence rate of 5%–19% after MWA in NSCLC with tumor <4 cm in a systemic review. It has been reported that thermal ablation combined with TACE can decrease the recurrence rate and may improve the prognosis compared to ablation monotherapy for early-stage liver cancer (18, 19). Identical results were demonstrated in the combination therapy of DEB-TACE and MWA compared to MWA monotherapy for early-stage liver cancer (20). A study from China reviewed 138 advanced NSCLC patients and found that the 1-, 2-, and 3-year survival rates of the combination therapy group (BACE and radiofrequency ablation) were 90.7%, 58.1%, and 20.9%, respectively, which revealed a better prognosis than that of BACE or ablation monotherapy groups (41). In this study, the combination therapy of MWA and DEB-BACE revealed a superior local control, with a higher 6-month PFS rate and a longer PFS than DEB-BACE alone. The potential mechanisms were inactivating the majority of tumor tissues that can be achieved directly by MWA, and DEB-BACE leads to the continuous delivery of loaded chemotherapeutic drugs and the embolization of tumor-feeding arteries, thereby reducing the collateral circulation and enhancing the synergistic anticancer effects on residual tumor tissue. Although the combination therapy reveals a trend of prolonging the OS, no significant difference was found. The leading causes were that 42.9% of the patients in group A survived until the last follow-up and the limited mean follow-up of 21.7 ± 14.1 months in this study.

This study has several limitations. First, it was a retrospective study; patient selection bias may exist. Second, the sample size was still limited for patients treated with combination therapy. Third, this study consists of advanced patients who are ineligible for or are refractory to standard treatments, and heterogeneity may exist. Fourth, the sequence of MWA and DEB-BACE is required to be further investigated. Fifth, the follow-up period was still limited, and the long-term evaluation of OS for combination therapy should be further explored.

In conclusion, the combination therapy of MWA and DEB-BACE is an effective and safe approach for ASTRI-NSCLC. MWA sequentially combined with DEB-BACE was superior to DEB-BACE alone in the local control of ASTRI-NSCLC. Although the combination therapy reveals a trend of prolonging OS, long-term prognosis warrants an investigation with a longer follow-up.

## Data Availability Statement

The data used to support the findings of this study are available from the corresponding author upon request.

## Ethics Statement

The studies involving human participants were reviewed and approved by Beijing Hospital, National Center of Gerontology, Institute of Geriatric Medicine, Chinese Academy of Medical Sciences. The ethics committee waived the requirement of written informed consent for participation. Written informed consent was obtained from the individual(s) for the publication of any potentially identifiable images or data included in this article.

## Author Contributions

SX: conceptualization, data curation, methodology, and writing—original draft. ZXB: data curation, methodology, resources, and writing—review and editing. YML: writing—review and editing. BL: resources and writing—review and editing. FLK: writing—review and editing. JZP: writing—review and editing. XGL: validation, supervision, and writing—review and editing. All authors contributed to the article and approved the submitted version.

## Funding

This work was funded by the Clinical and Translational Medical Research Fund, Chinese Academy of Medical Sciences (no. 2020-I2M-C&T-A-021). Funding source had no involvement in the conduct of the research and preparation of the article.

## Conflict of Interest

The authors declare that the research was conducted in the absence of any commercial or financial relationships that could be construed as a potential conflict of interest.

## Publisher’s Note

All claims expressed in this article are solely those of the authors and do not necessarily represent those of their affiliated organizations, or those of the publisher, the editors and the reviewers. Any product that may be evaluated in this article, or claim that may be made by its manufacturer, is not guaranteed or endorsed by the publisher.
